# Anchoring ZnO Nanoparticles in Nitrogen-Doped Graphene Sheets as a High-Performance Anode Material for Lithium-Ion Batteries

**DOI:** 10.3390/ma11010096

**Published:** 2018-01-10

**Authors:** Guanghui Yuan, Jiming Xiang, Huafeng Jin, Lizhou Wu, Yanzi Jin, Yan Zhao

**Affiliations:** 1 School of Chemistry and Chemical Engineering, Ankang University, Ankang 725000, China; chem_yuan@163.com (G.Y.); jimingxiang@aku.edu.cn (J.X.); hfjin5155@126.com (H.J.); akxywlz@163.com (L.W.); streamlet2000@163.com (Y.J.); 2 Synergy Innovation Institute of GDUT, Heyuan 517000, China

**Keywords:** anode material, graphene nanocomposite, lithium-ion battery, ZnO/nitrogen-doped, ZnO nanoparticles

## Abstract

A novel binary nanocomposite, ZnO/nitrogen-doped graphene (ZnO/NG), is synthesized via a facile solution method. In this prepared ZnO/NG composite, highly-crystalline ZnO nanoparticles with a size of about 10 nm are anchored uniformly on the N-doped graphene nanosheets. Electrochemical properties of the ZnO/NG composite as anode materials are systematically investigated in lithium-ion batteries. Specifically, the ZnO/NG composite can maintain the reversible specific discharge capacity at 870 mAh g^−1^ after 200 cycles at 100 mA g^−1^. Besides the enhanced electronic conductivity provided by interlaced N-doped graphene nanosheets, the excellent lithium storage properties of the ZnO/NG composite can be due to nanosized structure of ZnO particles, shortening the Li^+^ diffusion distance, increasing reaction sites, and buffering the ZnO volume change during the charge/discharge process.

## 1. Introduction

Lithium-ion batteries (LIBs) are one of the most promising modern electrochemical devices for energy storage, due to their high voltage, high energy density, and long lifespan [1,2,3,4]. Graphite has been widely used as the anode material due to its exhibiting stable performance in commercial LIBs [5]. Whereas the theoretical capacity of graphite is only 372 mAh g^−1^ and it cannot meet the increasing power demands [6]. To address these issues, remarkable efforts have been made to develop various promising candidates for anode materials to replace graphite, such as transition metal oxides (MO, M = Mn, Fe, Co, Ni, Cu, Zn, etc.) [7,8,9,10,11,12,13,14].

Among the above metal oxides, ZnO anode stands out as a potential alternative anode due to its high theoretical capacity (978 mAh g^−1^), low cost, ease to preparation, and chemical stability [15,16,17]. However, pure ZnO generally exhibits low reversible capacity and severe capacity fading, such a result is mainly caused by its large volume variation during the Li-ion insertion/extraction processes [16]. A lot of effort has therefore been devoted to conquer the above-mentioned shortcomings and a series of methods have been performed to improve properties of ZnO electrode. These methods include (i) preparing ordered ZnO nanostructured materials [18,19,20,21]; (ii) compositing ZnO with carbon materials [5,15,16,17]; (iii) doping with other metal oxides [22,23,24]. These techniques can improve conductivity, promote the lithiation/delithiation process, or buffer volume changes to mitigate the pulverization of the active particles. Among these methods, the remarkable and effective strategy to overcome the low conductivity is to composite ZnO with high electro-conducting material such as porous carbon in the form of carbon supporting [25]. Among various carbon materials, graphene has been recognized as very serviceable material because of its excellent high thermal conductivity, electrical conductivity, and high specific surface area [26]. Considerable efforts to achieve controllable graphene and ZnO/graphene composites have been extensively made by many different techniques, such as plasma synthesis [27], homogenizing dispersion method [28], stepwise heterocoagulation method [29], freeze drying method [30] and so on [31,32]. Although these composites demonstrated enhanced electrochemical performance, it is still not completely satisfactory. To meet this challenge, nitrogen-doped (N-doped) graphene can increase its conductivity by raising the Fermi level towards the conduction band compared with pure graphene [33]. Moreover, N-doped graphene (NG) can offer more active nucleation sites, which effectively prevent the aggregation of nanoparticles and strengthen the binding energy [34,35]. Therefore, compared to graphene, NG is more favorable for the composition of the nanosheets with metal oxides.

To the best of our knowledge, there are few studies on synthesizing ZnO/N-doped graphene composite. In this work, we report a facile and scalable method to synthesize nanostructured ZnO/N-doped graphene (ZnO/NG) nanocomposite. The effect of N-doped graphene on ZnO anode performance are systemically investigated in LIBs, in which the ZnO/NG nanocomposite can exhibit superior cycling stability and rate capability.

## 2. Results and Discussion

**Figure 1:**
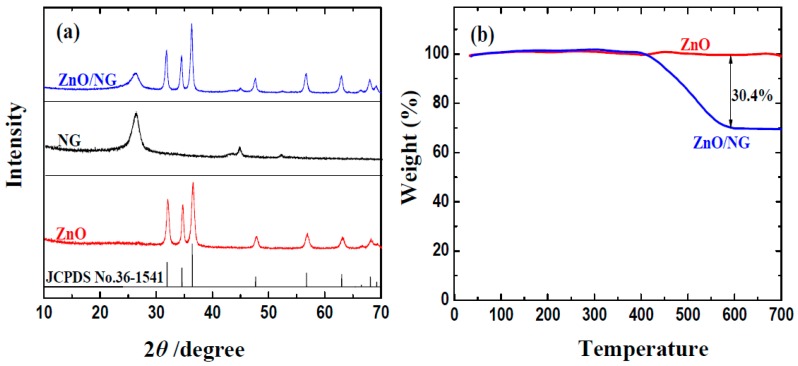
(**a**) XRD patterns; (**b**) thermogravimetric curves of ZnO and ZnO/NG composite.

The crystal structure of the obtained ZnO/NG composite is firstly investigated by XRD. Figure 1a exhibits the XRD patterns of ZnO, N-doped grapheme, and the as-prepared ZnO/NG composite. The main peaks of ZnO/NG composite agree with the peaks of ZnO, which can be indexed as ZnO with the lattice parameters of a = 0.325, b = 0.325, and c = 0.5207 nm (JCPDS No. 36-1451). In addition, the ZnO/NG composite displays an obvious diffraction peak at around 26°, which is attributed to the (002) reflection of stacking layers of graphene. The average size of ZnO crystallite in ZnO/NG composite is calculated to be 9.9 nm using the Debye Scherrer’s formula. The XRD pattern of ZnO/NG indicates that small ZnO nanoparticles are successfully deposited on the surface of N-doped graphene sheets. The presence of N-doped graphene has no significant effect on the formation of ZnO nanoparticles. The results can also be proved by the following TEM data. The successful deposition of ZnO nanoparticles on the surface of N-doped graphene sheets can facilitate electron transportation and alleviate the volume expansion of ZnO during the discharge/charge cycling, resulting in a drastic improvement of the electrochemical performance, which can be proved by the following electrochemical measurements. To determine the percentage of ZnO in the prepared ZnO/NG composite, thermo gravimetric (TG) measurement was carried out under an air atmosphere. As shown in Figure 1b, the TGA curve of ZnO/NG has an obvious one-step weight loss from around 400 °C. When the temperature reaches 700 °C, the sample remains approximately unchanged. Comparing to the TGA curve of pristine ZnO, the percentage of coating NG in ZnO/NG composite is about 30.4 wt %.

**Figure 2:**
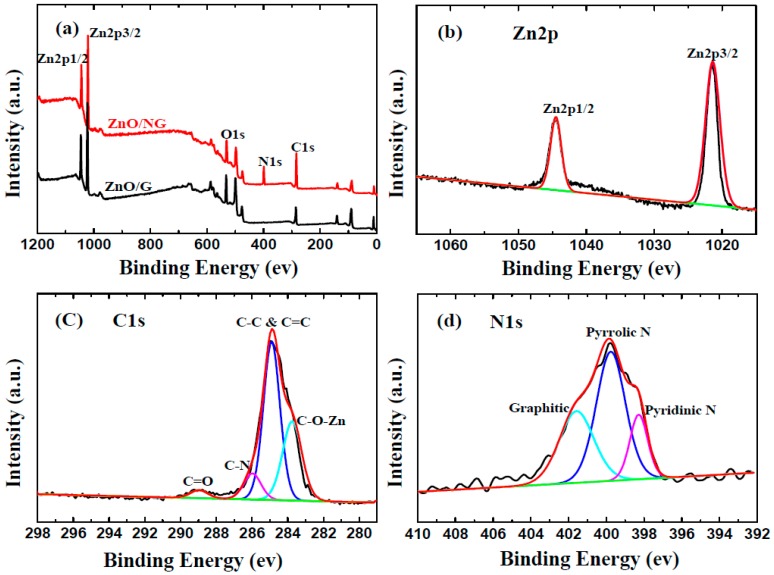
Survey XPS spectra (**a**) of ZnO/NG and ZnO/G composites; XPS spectra of (**b**) Zn2p, (**c**) C1s, and (**d**) N1s of ZnO/NG composite.

To elucidate the valences of C, Zn, N elements and their bonds configuration in ZnO/NG composite, XPS measurements are further investigated. Figure 2a displays the survey spectrums of ZnO/NG and ZnO/G composite. Four elements (Zn, O, C, and N) can easily be detected through the characteristic peaks of Zn2p, O1s, C1s, and N1s in the survey spectrums of ZnO/NG composite. In comparison, only the Zn, C, and O elements are identified through the survey spectrum of ZnO/G composite. The result reveals that nitrogen doping existed in the ZnO/NG composite synthesized by our simple solution method. Figure 2b shows the high-resolution XPS spectra of Zn2p, which consists of two strong peaks at 1045.5 eV and 1022.5 eV, identifying the Zn2p1/2 and Zn2p3/2 spin-orbit peaks of ZnO, respectively. The Zn2p1/2−Zn2p3/2 energy separation is around 23.0 eV. The high-resolution scan of C1s is shown in Figure 2c. The strongest peak at 285.0 eV corresponds to the graphite-like sp2 C (C−C and C=C bonds), indicating that most C atoms in the ZnO/NG composite are ranged in the conjugated honeycomb lattice. The peak located at 286.1 eV is related to the C-N linkage, which is originated from the N atoms’ incorporation of the ZnO/NG composite. The weakest peak at 288.8 eV reflects the C=O linkage, which indicates a little residual oxygen existed in the ZnO/NG composite. In addition, the special peak at 283.5 eV in the C1s spectrum can be assigned to the Zn-O-C bond [36]. The N1s spectrum of the ZnO/NG composite shown in Figure 2d can be attributable to the pyridinic N, pyrrolic N, and graphitic N atoms doped in graphene, according to the peaks at 398.3, 399.8, and 401.7 eV, respectively [37,38]. The N atoms doped in graphene have excellent electron donor characteristics and good charge mobility in the graphene lattice, which can effectively improve carbon catalytic activity in electrochemical reactions [39]. Predicatively, the ZnO/NG composite obtained in this work can enhance the electrochemical performance of anode material in lithium-ion batteries.

**Figure 3:**
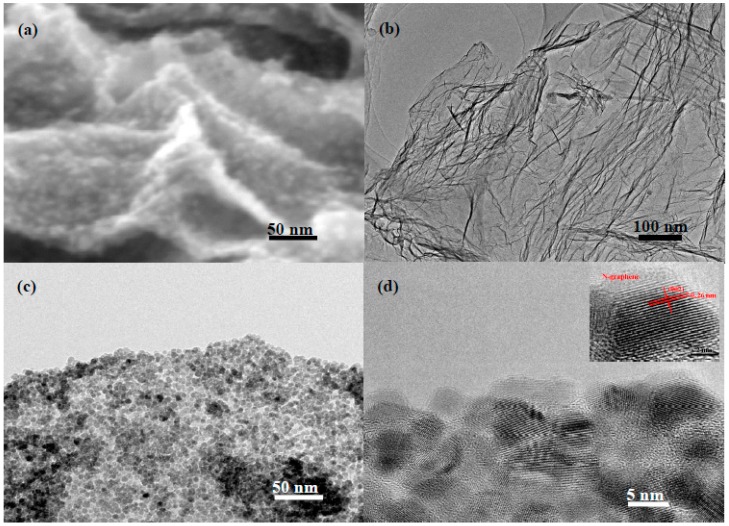
(**a**) SEM image of the ZnO/NG composite; TEM images of (**b**) NG and (**c**,**d**) ZnO/NG composite.

Figure 3 shows the scanning electron microscopy (SEM) and transmission electron microscopy (TEM) images of the as-prepared NG and ZnO/NG composite. The SEM image shows that small ZnO nanoparticles were well supported homogeneously on the curved N-doped graphene surface, as seen in Figure 3a. To further investigate the distribution and size of ZnO particles in ZnO/NG composite, the pristine NG and ZnO/NG composites are characterized by TEM. Figure 3b shows the TEM image of the synthesized N-doped graphene. The stacking layers of N-doped graphene show an obvious wrinkled surface, which is beneficial for ZnO nanoparticles to deposit and anchor on. As shown in the high-magnification TEM image of ZnO/NG composite (Figure 3c), small ZnO nanoparticles are barely obvious and agglomeration and the nanoparticles size are homogeneous. This result strongly indicates that the presence of nitrogen plays an essential role in the formation of homogeneous ZnO. The ZnO nanoparticle diameter distribution obtained from TEM is determined to be about 10.0 nm (Figure 3d). The lattice fringe of an interplane distance is measured to be 0.26 nm, which corresponds to the (002) plane of the ZnO crystals (insert in Figure 3d). The SEM and TEM images prove that the ZnO/NG nanocomposite is successfully synthesized by our facile method. In the prepared ZnO/NG nanocomposite, the ZnO particles with a size of about 10.0 nm are anchored uniformly on the wrinkled and twisted N-doped graphene sheets.

**Figure 4:**
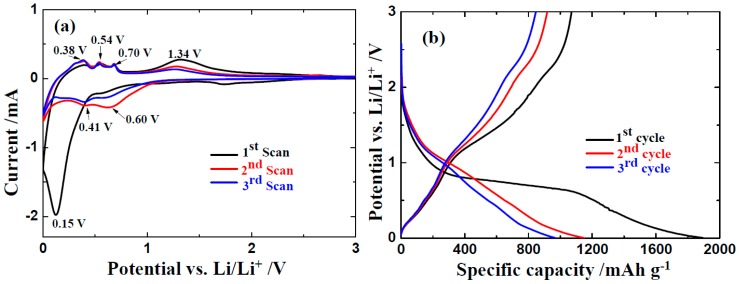
(**a**) CV curves of the ZnO/NG composite electrode at a scan rate of 0.1 mV s^−1^ and (**b**) discharge/charge profiles of ZnO/NG anode at 100 mA g^−1^.

To examine the effect of N-doped graphene, the electrochemical performance of ZnO/NG nanocomposite in lithium-ion battery is systematically investigated. The cyclic voltammogarm (CV) profiles of ZnO/NG electrode in the initial three cycles are exhibited in Figure 4a at 0.1 mV s^−1^ between 0–3.0 V. In the first cathodic scan, a strong reduction peak nearby 0.15 V can be observed in the ZnO/NG composite electrode. It could be assigned to the reaction of ZnO with Li into Zn and Li_2_O, the formation of Li-Zn alloy, and the solid electrolyte interphase (SEI) layer. In the subsequent second and third cathodic scans, the reduction peaks nearby 0.41 and 0.60 V demonstrate the reversibility of the lithiation process of ZnO. The disappearance of the strong peak nearby 0.20 V indicates that the formation of the SEI layer is irreversible. The initial three anodic scans exhibit consistency in shape. The oxidation peaks nearby 0.38, 0.54, and 0.70 V indicate the multi-step decomposition of Li–Zn alloy, while the nearby 1.34 V can be ascribed to the decomposition of Li_2_O and formation of ZnO with the reaction between Li_2_O and Zn [18,20]. Overall, the CV curves show high reproducibility and consistency, indicating good reversibility of the ZnO/NG electrode. The intensity of reduction and oxidation peaks grow weaker with the scans progress, which may be because of the incomplete conversion between Zn and ZnO. Figure 4b depicts the initial galvanostatic charge/discharge profiles of the ZnO/NG nanocomposite at a current density of 100 mA g^−1^. As shown in Figure 4b, the plateau voltages of the charge/discharge profiles are in good agreement with the peak voltages in CV profiles in Figure 4a. The ZnO/NG nanocomposite delivers a large discharge capacity of 1894 mAh g^−1^ in the first cycle. The coulombic efficiency is only 56.5%, which is mainly caused by the irreversible extra discharge capacity during the SEI layer formation. The long slope region observed in the initial discharge disappears in the following cycles. The ZnO/NG nanocomposite delivers discharge capacities of 1148 and 968 mAh g^−1^ in the second and third cycles, respectively. The corresponding coulombic efficiencies have reached points of 79.9% and 87.4%. After the first discharge process, the charge/discharge curves maintain a similar size and shape, indicating the ZnO/NG nanocomposite keeps a relatively steady state during the lithiation/delithiation processes.

**Figure 5:**
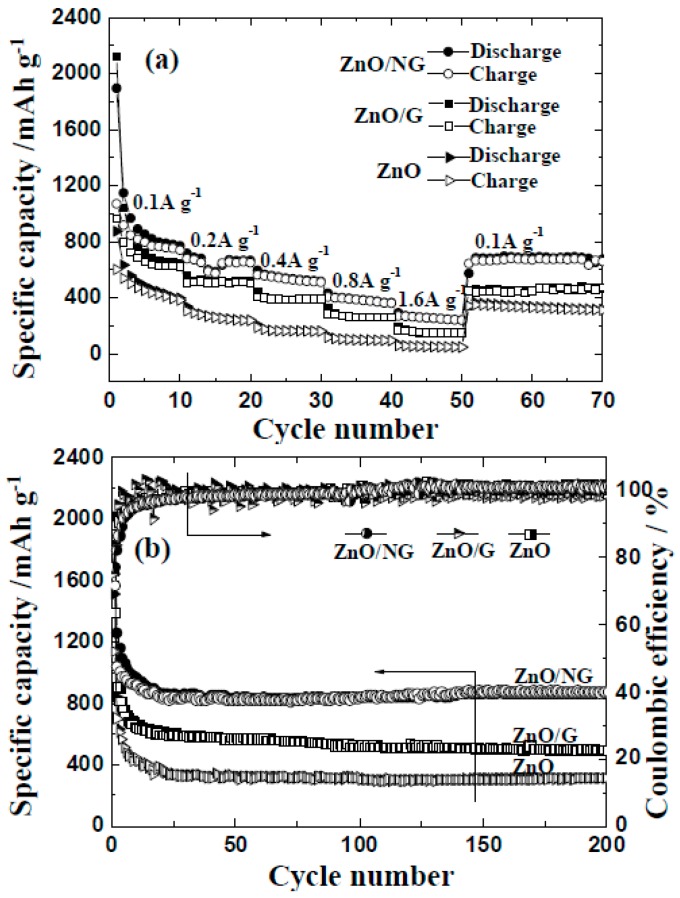
(**a**) Rate capabilities; (**b**) cycle abilities of ZnO, ZnO/G, and ZnO/NG anodes at 100 mA g^−1^.

The rate performance and cycle ability are important aspects for the application of ZnO/NG nanocomposite in lithium-ion batteries. Figure 5a shows the rate performances of the ZnO/NG composite and its counterparts (pristine ZnO and ZnO/G composite) at 100 mA g^−1^, respectively. The ZnO/G composite is prepared by the same procedure as preparing ZnO/N-doped grapheme, just using graphene instead of N-doped graphene. So the influence of different experiment methods on different electrochemical performances can be eliminated. As demonstrated in Figure 5a, the test current increases stepwise, and the rate performances of the ZnO/NG and ZnO/G electrodes have been significantly improved comparing to the pristine ZnO electrode. In detail, for the ZnO/NG electrode, the reversible discharge capacities of 968, 687, 567, 404, and 271 mAh g^−1^ are achieved at current rates of 100, 200, 400, 800, and 1600 mA g^−1^, respectively. The further return of the discharge rate to the initial 100 mA g^−1^ can recover the stable reversible discharge capacity of nearly 680 mAh g^−1^. For the ZnO/G electrode at different current densities of 100, 200, 400, 800, and 1600 mA g^−1^, the reversible discharge capacities of each period are 849, 521, 404, 282, and 166 mAh g^−1^, respectively. While for the pristine ZnO electrode, the reversible discharge capacities at each current rate are 100~300 mAh g^−1^ smaller than that of the ZnO/G and ZnO/NG electrodes. Long-term cycle abilities of the ZnO, ZnO/G, and ZnO/NG anodes at 100 mA g^−1^ are exhibited in Figure 5b. It can be easily seen that both the specific capacities and capacity retentions of the ZnO/G and ZnO/NG anodes are much better than that of the ZnO anode. During the three anodes, the ZnO/NG anode could maintain the highest reversible specific discharge capacity of 870 mAh g^−1^ at 100 mA g^−1^ after 200 cycles. The ZnO/G and pristine ZnO anodes could only deliver the reversible specific discharge capacity of 493 and 318 mAh g^−1^ at 100 mA g^−1^ after 200 cycles, respectively. The coulombic efficiencies of the three anodes are a little lower in the first several cycles, which could be inferred from the irreversible extra discharge capacity during the SEI layer formation. After 50 cycles, all the coulombic efficiencies of these anodes stabilize around 99%, showing good capacity retention. Detailed observations from Figure 5 reveal that the reversibility and cycling stability of the ZnO/NG and ZnO/G anodes are better than that of the pristine ZnO anode. These enhanced electrochemical performances may be due to the positive effect of the graphene or N-doped graphene additive, which are not only capable of providing a high electronic conductivity and short Li^+^ diffusion distances, but also serving as a stable carrier for ultrafine ZnO particles anchored on it. Furthermore, the ZnO/NG nanocomposite can deliver better electrochemical performances than ZnO/G composite, which may be originated from the following two aspects. Firstly, the N atoms doped in graphene have excellent electron donor characteristics and good charge mobility in graphene lattice, which can effectively improve carbon catalytic activity in electrochemical reactions [33,34]. Secondly, the binding energy between ultrafine ZnO particles and N-doped graphene nanosheets are improved due to the extra active sites provided by nitrogen doping.

Compared to previous work, the as-prepared ZnO/NG composite exhibits more excellent electrochemical performances, as listed in Table 1. The large initial discharge capacity delivered by ZnO/NG composite is mainly caused by the irreversible extra discharge capacity during the SEI layer formation. The significantly improved cycle ability of ZnO/NG composite benefits from the unique structure and the positive effect of the N-doped graphene. The N-doped graphene nanosheets can not only improve the conductivity of the ZnO/NG composite and provide more active electrochemical sites, but they can also alleviate the volume expansion of ZnO during the discharge/charge cycling. It is worth noting that N-doped graphene itself can be used as anode material for lithium-ion batteries. During the discharge process of the ZnO/NG composite, some Li ions can react with ZnO to form Li-Zn alloy, other Li ions can intercalate into the stacking layers of N-doped graphene in the meantime. Therefore, the ZnO/NG composite shows better lithium storage capacities than N-doped graphene material.

**Figure 6:**
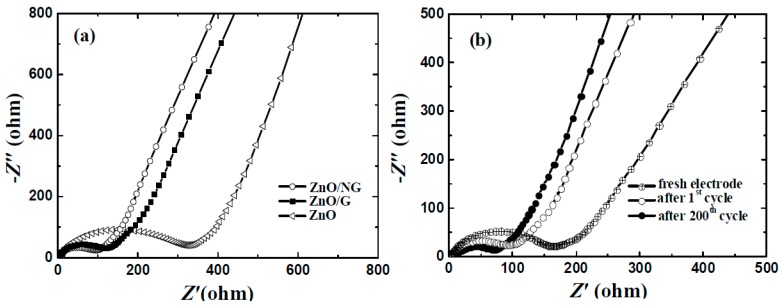
Nyquist plots of (**a**) ZnO, ZnO/G and ZnO/NG electrodes after first discharge/charge cycle; and (**b**) ZnO/NG electrode at different stages with the frequency region of 100 kHz to 0.01 Hz.

To confirm the positive effect of graphene or N-doped graphene additives on the conductivity and charge transfer behavior in composites, electrochemical impedance spectroscopy (EIS) measurements were investigated on the ZnO, ZnO/G, and ZnO/NG electrodes. The EIS data for the three electrodes measured after the first cycle are shown in Figure 6a. All the impedance plots for the fully discharged states are composed of two parts, a semicircle in high-to-medium frequency and a straight line in low frequency. The semicircle is mainly attributed to the charge transfer impedance of the electrode. The straight line is mainly attributed to the Warburg impedance reflecting the solid-state diffusion of Li^+^ into the bulk of the active materials [40]. It can be easily seen from Figure 6a that the charge transfer impedances of the ZnO, ZnO/G, and ZnO/NG electrodes are nearly 325, 130, and 90 Ω, respectively. Considering the formation of SEI is completed after first cycle, the impedances can be mainly attributed to the additive and the material structure. By adding graphene and N-doped graphene, the charge transfer impedances of the ZnO/G and ZnO/NG electrodes decrease dramatically, indicating that the graphene or N-doped graphene additive plays a significant role in improving the conductivity. The ZnO/NG electrodes exhibit the smallest charge transfer impedance. The results could be due to the changes in the material conductivity and morphology introduced by N-doped graphene sheets [35]. The N-doped graphene can enhance the charge transfer conditions by providing an effective electron conduction path and shortening the Li^+^ diffusion distance within the nanosized ZnO particles anchored evenly on the N-doped graphene sheets. In addition, the EIS data of the ZnO/NG electrode at different stages are shown in Figure 6b. The diameter of semicircles in the high-to-medium frequency region reduce gradually as the test proceeds, indicating the decrease of the charge transfer impedances. The charge transfer impedance of the new ZnO/NG electrode is as high as about 170 Ω, which is mainly because the effective electron conduction paths and SEI layer are not formed in fresh electrode. Low and stable charge transfer impedances can be realized for the ZnO/NG electrode during repeated discharge/charge cycles, which is about 90 Ω and 80 Ω after 1st cycle and 200th cycle, respectively. This phenomenon indicates that the special structure of the ZnO/NG composite has beautiful rigidity and stability to endure the repeated lithium insertion/extraction processes.

## 3. Materials and Methods

Synthesis of nitrogen-doped graphene (NG): Firstly, graphene oxide (GO) was synthesized from natural flake graphite by a modified Hummers’ method [41]. Subsequently, 0.060 g GO was dispersed into 100 mL ethanol. After ultrasonication for 2 h, 12 mL hydrazine hydrate and 8 mL of ammonia (25%) were added into the suspension in order. The mixture was stirred vigorously for 15 min and then sealed into three 50 mL teflon-lined stainless steel autoclaves at 180 °C for 3 h. The black powder in autoclaves was collected and washed by ethanol and distilled water and then dried in a vacuum oven at 80 °C overnight to obtain the NG product.

**Figure 7:**
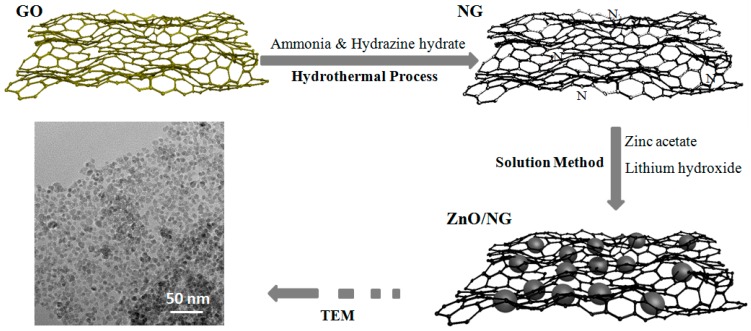
The preparation of the ZnO/NG composite.

Synthesis of ZnO/NG composite: Firstly, 0.720 g Zinc acetate (Zn(CH_3_COO)_2_) and 0.020 g NG were dispersed in 50 mL ethanol to form a homogeneous solution. A measure of 0.190 g lithium hydroxide (LiOH) was dissolved in another 50 mL ethanol solution. Both solutions were stirred for 2 h using a magnetic stirrer. Subsequently, the LiOH solution was added dropwise to the Zn(CH_3_COO)_2_ solution under constant vigorous stirring. After stirring for 24 h at room temperature, a homogenously dispersed black suspension was obtained. The black powders were separated via filtration and washed thoroughly by deionized water and ethanol. Finally, the black ZnO/NG composite was obtained followed by freeze-drying in vacuum freeze drier and grinding in agate mortar. For comparison, a ZnO/G sample with addition of graphene and a ZnO sample without any addition were prepared as well by the same procedure. The preparation of the ZnO/NG composite is illustrated in Figure 7.

Powder X-ray diffraction (XRD, SmartLab, Rigaku Corporation, Tokyo, Japan) patterns of the samples were detected using Cu Kα radiation (λ = 0.15418 nm) at 0.02° s^−1^ in the 2θ range of 10°–70°. Scanning electron microscopy (SEM) and transmission electron microscopy (TEM) images were obtained on a Quanta 400 ESEM-FEG instrument (FEI Corporation, Hillsboro, OR, USA) and a JEM-2100F instrument (JEOL Corporation, Akishima, Tokyo, Japan), respectively. X-ray photoelectron spectroscopy (XPS, PHI 5400 electron spectrometer) was performed using unmonochromated Mg Kα radiation (hν = 1253.6 eV) as the excitation source. NG content in ZnO/NG composite was estimated by thermogravimetric analysis (TGA, SDT Q600, TA Instruments, New Castle, DE, USA) in the temperature range of 30–700 °C (10 °C/min) under air atmosphere.

The testing ZnO/NG, ZnO/G, and ZnO electrodes were prepared by mixing 80 wt % as-prepared active materials, 10 wt % polyvinylidene fluoride (PVDF, Sigma-Aldrich Corporation, Milwaukee, WI, USA), and 10 wt % acetylene black in *N*-methylpyrrolidone (NMP, Sigma-Aldrich Corporation, Milwaukee, WI, USA), and 10 wt % acetylene black in) solvent to form homogeneous slurries. The mixture slurries were coated onto nickel foams measuring 10 mm in diameter and then dried in a vacuum oven at 120 °C for 10 h. In order to acquire preferable contact between active materials and nickel foams, these testing electrodes were pressed at 15 MPa for several minutes. The loading density of active material in each electrode was about 2 mg cm^−2^. For testing the electrochemical performances of the testing electrodes, the ZnO/NG//Li batteries were assembled in coin-type (CR2025) cells in a glove box filled with argon, using pure lithium foil electrodes as the counter electrodes. The electrolyte was a mixed solvent of diethylene carbonate, dimethyl carbonate, and ethylene carbonate (1:1:1) containing of 1 M LiPF_6_, and the separator was microporous polypropylene membrane (Celgard 2400). The testing ZnO/G and ZnO electrodes were prepared in the same way as the counterparts.

The galvanostatic charge and discharge tests were carried out on a program-control battery system (Wuhan LAND Electronic Co., Ltd., Wuhan, China). Cyclic voltammetry (CV) tests were performed on a electrochemical workstation (CHI 600E) at 0.1 mV s^−1^ in 0–3.0 V. Electrochemical impedance spectroscopy (EIS) properties were measured in the frequency range of 0.01 Hz–100 kHz using the same CHI 600E electrochemical workstation.

## 4. Conclusions

A novel ZnO/nitrogen-doped graphene (ZnO/NG) nanocomposite was synthesized via a facile solution method. In the prepared ZnO/NG nanocomposite, the ZnO particles with size of about 10.0 nm were anchored uniformly on the wrinkled and twisted N-doped graphene sheets. When used as anode materials, the ZnO/NG nanocomposite exhibited much better electrochemical abilities compared to pristine ZnO and the ZnO/graphene (ZnO/G) nanocomposite. The resulting ZnO/NG nanocomposite can maintain a reversible specific discharge capacity at 870 mAh g^−1^ after 200 cycles at 100 mA g^−1^. The enhanced electrochemical performances of the ZnO/NG nanocomposite can be attributed to the N-doped graphene additive and the unique structure of the composite. The cross-linked N-doped graphene nanosheets in the ZnO/NG nanocomposite have outstandingly improved conductivity and high surface areas, which facilitate electron transportation and provide plenty of active sites for lithium ions, resulting in a drastic improvement of the rate performance. The ultrafine ZnO particles anchored evenly on N-doped graphene nanosheets can not only enlarge the electrolyte/ZnO contact area, but also alleviate the volume expansion of ZnO during the discharge/charge cycling, resulting in an extreme enhancement of the cycle stability.

## Figures and Tables

**Table 1:** Comparison of electrochemical performances between the N-doped graphene and ZnO/NG composite.

Sample	Initial Discharge Capacity	Cycle Number	Rate	Reversible Capacity	Ref.
ZnO/NG	1894 mAh g^−1^	200	100 mA g^−1^	870 mAh g^−1^	this work
ZnO@graphene	1050 mAh g^−1^	50	1 C	460 mAh g^−1^	[28]
Graphene-porous carbon-ZnO	1300 mAh g^−1^	100	0.1 C	660 mAh g^−1^	[29]
ZnO–graphene	1450 mAh g^−1^	100	100 mA g^−1^	900 mAh g^−1^	[30]
N-doped graphene	1420 mAh g^−1^	50	100 mA g^−1^	630 mAh g^−1^	[39]
